# Strength of bed nets as function of denier, knitting pattern, texturizing and polymer

**DOI:** 10.1186/1475-2875-10-87

**Published:** 2011-04-14

**Authors:** Ole Skovmand, Rune Bosselmann

**Affiliations:** 1 Intelligent Insect Control, 118 Ch Alouettes, Castelnau le Lez, France

## Abstract

**Background:**

Bursting strength is a standard method for evaluating mosquito net strength. This article suggests that tension strength with one grab and one hook better represent how holes are generated in bed nets in real life.

**Methods:**

Measurements of bursting strength and tension strengths in the two directions are analysed for eight model nets created for the study. The nets were made in the most commonly used denier (75 and 100 D) and mesh (156 holes/inch^2^) for multifilament polyester yarns, texturized or not, and with 4 or 6 sided holes. All were made from one polyester quality. Data was arranged in a randomized, complete block design and analysed for significant variables and their interactions. Data was then subjected to regression analyses using net square metre weight as a weighting factor with stepwise removal of variables. This revealed how the four textile variables interacted and allowed for making predictions for the strength of commercial nets in polyester or polyethylene.

**Results:**

For the model nets, higher denier provided higher bursting strength and tension strengths, texturizing weakened nets and four-sided holes were stronger than six-sided holes. Even when compensating for square metre weight, 100 D nets are stronger than 75 D nets. Results for the commercial polyester net nets are less clear, probably because of different qualities of polyester. Tensile strength: a 75 denier net knitted tightly to provide the same square metre weight as a standard 100 denier net therefore does not obtain the same strength. Polyethylene nets are made of mono-fibre yarns and, therefore, have higher tension strength in both directions than multifilament polyester nets. For bursting strength results overlap for 100 denier yarns of both yarn types. As a class, commercial polyethylene nets are stronger than commercial polyester net whatever method is used for evaluation.

**Conclusion:**

Tension strength measured in the length and width directions of the net using one hook and one clamp provide new relevant data as this method more closely imitates the cause of tear holes in nets as they occur in real life use. Using this methodology, the commercial monofilament yarn polyethylene nets are significantly stronger than the commercial multifilament polyester nets. This test method should be applied for nets used for years in the field.

## Background

The long-lasting insecticide-treated mosquito net (LLIN) is one of the major tools against malaria in the current anti-malarial campaigns. All nets currently recommended by the World Health Organization (WHO) use a pyrethroid as the insecticide. The effect of these nets over time depends on the loss rate of insecticide and textile integrity. A holed net provides reduced protection or no protection for the person sleeping under the net [1,2]. Presently, there are very few studies on the physical longevity of bed nets that take into consideration the percentage of nets discarded by the users during the study. Since only the nets which the users considered useful are included in the study, most investigations overestimate the span of time during which bed nets are used [3]. Furthermore, among the studies of multifilament polyester nets, no clear distinction has been made between 75 and 100 denier (D) net, so it is not known if one is more persistent than the other. In addition to polyester nets, WHO has also recommended three mono-filament high density polyethylene (HDPE) bed nets, one of which has been on the market since the mid-90s. A field study has indicated that one make of this net type was still sufficiently intact and active after seven years [4], where 100 out of 103 nets followed over the seven years were still in use.

A large-scale study was initiated by Centers for Disease Control and Prevention (CDC) in 2009 in which the nets that carried a WHO recommendation that year were to be followed for 3-5 years. Meanwhile, there are few reliable studies and data available is open to interpretation as demonstrated in recent WHO reports on the two first LLIN [5], which evaluated Permanet 2 and [6], and a claim that the Olyset Net remained effective for five years.

Therefore, there is a need to compare nets based on objective physical methods that can be related to events in the field. This study argues that a tensile strength test with one grab and one hook best imitates the cause for many net holes; when a net is caught on a pointed obstacle and then pulled by the user to get it free. Data from such studies and from studies on bursting strength are presented for various types of polyester net and polyethylene nets. To obtain more general knowledge on parameters for determining net strength, polyester nets made with yarns from a single producer were obtained. In this production, all process parameters were controlled from extrusion to knitting. This allowed for determining the exact effects of texturizing, denier and knitting pattern. Insights about these effects are then used to understand strength of commercial nets.

## Methods

### Definitions

Denier (D) represents the weight (g) of 9,000 metre yarn. Texturizing is a surface treatment that rips up the surface of a smooth yarn to create a softer yarn with a bigger surface. A texturized yarn is called Drawn Texture Yarn (DTY), whereas the smooth yarn is called Fully Drawn Yarn (FDY). In Raschel knitted textiles, here LLIN, all yarns are parallel and the denominations Length and Width are used for direction of the knitting and across the net, respectively.

### Nets

Polyester (PES) nets made for this study were produced in a polyester factory in Fuzhou, China and are referred to in the report as the model nets. This producer makes the polyester nets from extrusion of the yarn to texturizing, including knitting and heat setting. No nets were coloured. All parameters could therefore be standardized and nets were exposed to the exact same condition during extrusion, warping, knitting (though knitted in two ways, but on the same machines from Karl Mayer) and heat setting. One type of PES granules were used for all yarns. Yarns were extruded in 75 and 100 D with 36 filaments (as for all commercial LN nets). The yarns were either texturized or not. PES nets were Raschel knitted with a 156 mesh/inch^2 ^(25 holes/cm^2^) with rhomboid or hexagonal holes.

Commercial nets were bought from the market and sent in unopened sacks to the WHO reference centre for textile studies CITEVE (Centro Tecnologico das Industrias Textil e do Vestuario de Portugal). Two nets were made of 75 D texturized PES yarns (Permanet 2 (Vestergaard-Frandsen) and Interceptor (BASF)), one in 100 D texturized PES yarns (also a Permanet 2) and one, (Permanet 3) was made from 75 D texturized PES yarns but knitted in Atlas pattern [7], a method that makes the net more elastic. The lower part of the last net was knitted in a denser pattern than the upper part giving a square metre weight of approximately 40 and 30 g, respectively. Olyset Net Net (Sumitomo Chemical), Duranet (Clarke Mosquito Control) and Netprotect (Bestnet Europe ltd) old and current version nets are Rachel knitted with rhomboid holes, 25, 136 and 136 mesh/inch^2^, and 150 D, 150 D and 118 D, respectively.

### Measurements

Net measurements were carried out by the textile Institute CITEVE, which received the unused commercial nets in original packaging, and the other nets as large samples. CITEVE took samples from the intact nets according to the WHO protocol for net sampling, five per net, four from the sides following a diagonal and one from the roof. Approximately one square metre was sent for all other nets in the study.

**Figure 1:**
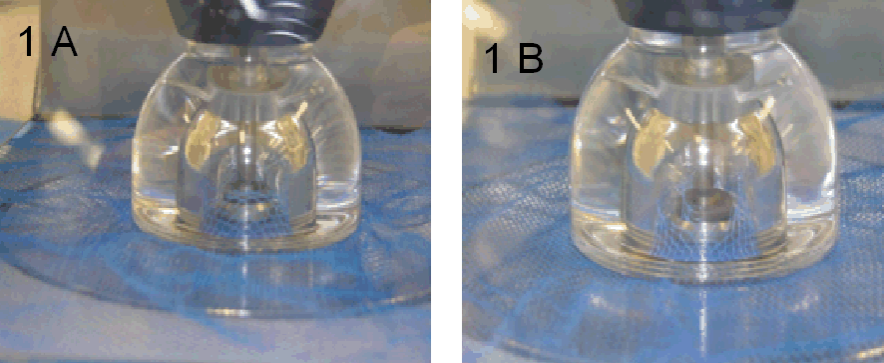
**(A and B). The bursting strength measuring apparatus**. The sample is clamped between upper and lower part and a pressure vertical to the surface until the net busts (Photo CITEVE).

Bursting strength was measured according to European Norm International Standards Organization (EN ISO) 13938-2. This test uses pneumatic pressure method for determining bursting strength and distension of textile fabrics before bursting (Figure 1A).

The test specimen is clamped over an expansive diaphragm by means of a circular clamping ring. Increasing compressed air pressure is applied to the underside of the diaphragm, causing distension on the diaphragm and the fabric. The pressure is increased smoothly until the test specimen bursts (Figure 1B) and the bursting strength (kPa) and bursting distension (mm) are recorded.

At the same conditions, the diaphragm is distended without the presence of the specimen by an equal amount to the mean height at burst of the test specimen. The pressure at this distension of the diaphragm is noted as "diaphragm pressure" and is subtracted to the pressure of the specimen. Five specimens were tested from each net or sample. Tensile strength with grabs was measured according to test standard ISO 13934-2 (tensile strength - grab method). This test determines the maximum force of textile fabrics using a grab method. The fabric specimen is gripped in its centre part by jaws of specified dimensions and is extended at constant rate until rupture. The maximum force was recorded.

**Figure 2:**
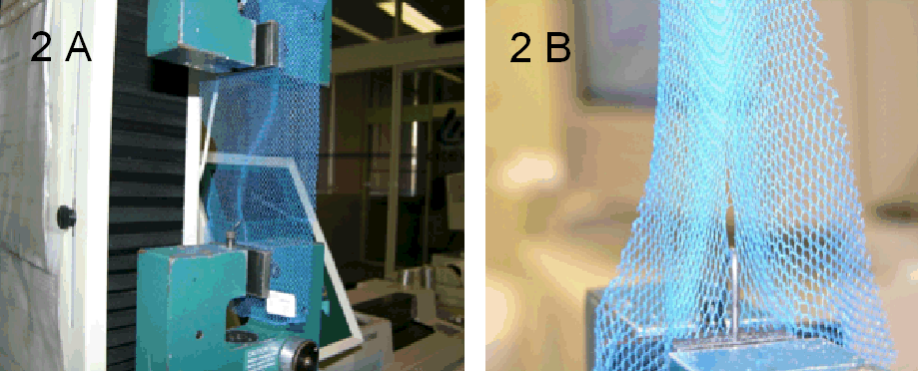
**(A and B). The tension strength apparatus**. The upper part of the sample is fixed in a clamp and the hook in a mesh hole of the net. The clamp draw until the net is torn at the hook (B) (Photo CITEVE).

Tensile strength with hook is an adaptation of the grab test, where a hook is positioned in one clamp (Figure 2A). During the test the hook is inserted in a mesh of the net. When the net is pulled, the hook will cause rupture in the net (Figure 2B). It thus determines the force necessary to tear a hole in the net.

The test is intended to imitate the daily handling situation of a net, where the net is caught on a pointed object (like a nail) when, e.g., putting the net away in the morning. Five specimens per net were tested in the length and width directions.

**Figure 3:**
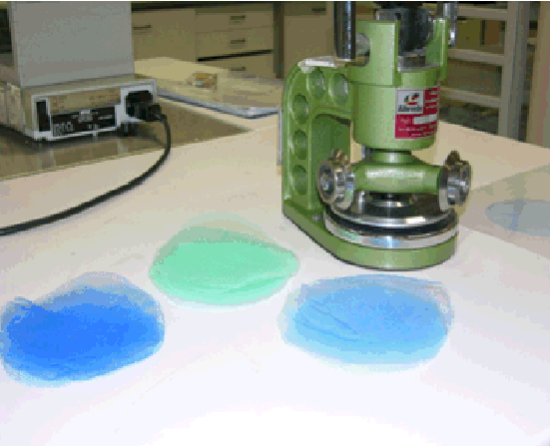
**Square metre weight apparatus**. The net is put on a table to rest for 24 hr and the circular cutter cuts a sample with a diameter of approximately 10 cm. The net weight is the average of several such circular cuttings taken according to WHOPES sampling procedure from bednets.

Fabric weight or mass per unit area was measured according to EN 12127 (Figure 3). The test is based on the measurement of small samples in the conditioned state. It is important to ensure that the fabric is in a relaxed state prior to testing. The fabric shall be kept in a flat tension-free state during conditioning (for at least 24 hours). Five test specimens with an area of 100cm^2 ^each are cut from the fabric and the mass of each specimen is determined by weighing.

Yarn diameters of PE nets were determined by microscope at 200 X amplification with an ocular metre scale. 20 determinations were made in different spots per net. Denier can be calculated from the diameter accepting that PE yarns has a specific gravity of 0,97 g/cm^3^.

Statistics: A randomized, complete block design was used to evaluate impact of the three independent parameters (denier, hole-shape and texture) and on the dependent parameters; net weight/m^2^, bursting strength and tensile strength in the direction width and length. In the statistical analysis, PES nets were given the value 1 and 0 for texturized (DTY) and smooth yarn (FDY), respectively. Analysis was carried out as a Factorial design using variance analysis (ANOVA) with three independent variables (denier, texturizing and hole shape) as variables. Pearson Correlation Analysis was used to guide a multi linear regression of all variables, dependent and independent. The multi linear regression analysis then showed which parameters had positive and which had negative effects on the dependent variables. Since square metre weight was influenced by several variables in combination, dependent variables were analysed in weighted least squares linear regression analyses with net weight/m^2 ^as weighting factor. This analysis revealed impact of these parameters beyond their impact on the net weight.

Simple variance analysis was used to compare means values of bursting strength of the different types commercialized polyester nets; Tukey test for significant difference was applied to contrast mean values. Statistix 9 for Windows was used [8].

## Results

Polyester (PES) nets: Five samples from each of the following net types were tested: (1) FDY yarn 75 Denier (D) knitted in rhomboid pattern, (2) FDY, 100 D, rhomboid; (3) FDY, 75 D, knitted in hexagonal pattern; (4) FDY, 100 D, hexagonal; (5) DTY, 75, rhomboid; (6) DTY, 100 D, rhomboid; (7)DTY, 75 D, hexagonal; and (8) DTY, 100 D, hexagonal. In Table 1, the rhomboid knitted nets are called four-holed and the hexagonal knitted nets are called six-holed. Table 1 shows average values of five repetitions.

**Table 1:** Strength values for model polyester nets

Sample	Weigth (g/m^2^)	Burst Strength (kPa)*	Tensile Strength with hook Length (N)§	Tensile Strength with hook Width (N)§
75 D 4 holed FDY	28,4	347 (7,8 mm)	42 (10%)	18 (5%)

100 D 4 holed FDY	40,1	449 (8,4 mm)	35 (11%)	26 (10%)

75 D 6 holed FDY	30,8	264 (9,5 mm)	38 (7%)	15 (5%)

100 D 6 holed FDY	41,3	320 (9,5 mm)	33 (14%)	22 (8%)

75 D 4 holed DTY	27,6	284 (6,8 mm)	24 (34%)	17 (8%)

100 D 4 holed DTY	36,9	384 (7,7 mm)	32 (12%)	21 (7%)

75 D 6 holed DTY	27,1	268 (8,3 mm)	40 (30%)	15 (13%)

100 D 6 holed DTY	37,7	304 (9,1 mm)	34 (10%)	22 (4%)

*The value in bracket is the distance the net was stretched before bursting. § the value in bracket is SD as a percentage of the mean.

Data was analysed in a factor design using ANOVA analysis having texture, hole shape and denier as variables. This analysis also allows for the examination of interactions between the independent variable in their effect on the dependent variables: square metre weight, bursting strength, how much the net gave in (mm) before bursting and the tensile strength in the two directions (Table 2). The analysis showed that weight was significantly dependent on yarn D, hole-shape and texturizing with significant interactions between the three independent variables. Bursting strength was significantly dependent on D, hole shape and texturizing, but there was an interaction effect of D X Hole shape and Hole Shape X texturizing. Height at bursting strength was significantly dependent on D, Hole Shape and texturizing. No interaction was significant. Tensile strength direction Length simply depended on D of yarn, but all 3 independent variables had combined effects. Finally, Tensile strength Width was significantly influenced by D of yarn and texturizing, but not of the shape of the hole. Only D x hole shape had a combination effect on tensile strength Width.

**Table 2:** Variation analysis of a complete randomized block design using Factorial design - model nets.

Variable	Denier		Hole Shape		Texturising		Denier X Hole Shape		Denier X Texturizing		Hole Shape X Texturizing	
Weight/m^2 ^(g)	657	<0.0001	28.1	<0.0001	47.2	<0.0001	11.2	0.0021	5.6	0.0238	8.7	0.0058

Bursting Strength (Pa)	87.8	<0.0001	100	<0.0001	13.0	0.0010	13.5	0.0008	1.0	0.95	16.7	0.0003

Height at Burst (mm)	33.3	<0.0001	154	<0.0001	40.6	<0.0001	0.04	0.84	2.1	0.16	0.02	0.88

Tensile strength Length	10.7	0.0025	0.24	0.6	0.0	0.95	13.8	0.0007	12.5	0.0013	20.4	0.0001

Tensile Strength Width	272	<0.0001	2.8	0.10	11.2	0.0019	3.8	0.0595	5.8	0.026	1.8	0.186

The first value per cell is the Error of Variance (F) value and the second the Probability (P) value. The first column shows the dependent variables and the following 3 columns how the independent variables influence the dependent. The last three columns analyze interactions between independent variables in their effect on the dependent variable.

The Pearson analysis showed that D, net weight/m^2^, bursting strength, length tension strength and knitting pattern were correlated. As shown in Table 1, texturizing the yarn resulted in a lower square metre weight and this effect was significant (Table 2). Table 2 shows that square metre weight depends on all three independent variable and all their possible interactions. When net weight/m^2 ^was used as a weighting factor, bursting strength was still correlated to Length tensile strength and D and negatively correlated to number of sides in the mesh (4 or 6). Tension strength direction Width was only correlated to texturizing (r^2^= 0.41).

Further analysis with multiple regressions used the net weight as a weighting factor, also based on the obvious idea that net square metre weight may indirectly impact strength. In this analysis variables were successively removed to find the variables determining strength.

Bursting strength: A linear, multi regression analysis using net weight/m^2 ^as weighting factor (Table 3) showed that burst strength is significantly correlated to D (P = 0.02) and hole shape (P = 0.01), but not significantly to texturizing (P = 0.14). When the variable hole shape was removed, the overall correlation coefficient declined, but results became more significant. Hole shape had a negative coefficient, meaning that holes with six sides gave lower bursting strength than holes with four sides.

**Table 3:** Linear multi regression analysis using weight/m^2 ^as weighting factor for the model nets

Variable/Regression	Constant	Denier		Hole Shape		Texturizing		Overall Correlation		
		**Coeff.**	**P**	**Coeff.**	**P**	**Coeff.**	**P**	**r**^**2**^	**F**	**P**

Bursting Strength	293	+ 2.94	0.02	-41	0.01	-35	0.14	0.89	11.3	0.020
	275	+2.95	0.03	-41	0.02			0.81	10.6	0.016

Height at Burst	3.32	+0.02	0.03	+0.70	0.001	-0.78	0.01	0.96	30.9	0.0032

Tensile Strength Length	39.7	-0.1	0.56	+ 1.2	0.59	-3.9	0.38	0.30	0.57	0.66

Tensile Strength Width	2.38	+0.26	0.004	-1.0	0.55	- 1.62	0.21	0.91	13.3	0.015
	-3.44	+0.26	0.003					0.78	22.0	0.003

Where the table presents two lines per row, the upper line includes all independent variables, whereas the lower line shows the significantly best fit after omitting variables that did not have significant impact.

Height at burst in the bursting strength test: When compensating for weight/m^2^, this variable was significantly correlated to all three independent variables and all had a positive effect (Table 3). The height that an FDY net stretched before bursting was greater than that of a matched DTY net.

Tensile strength in the Length direction using one clamp and one hook was not significantly dependent on any variable when square metre weight was used as a weighting factor (Table 3, P+0.64). When D and hole shape were removed from the regression as indicated in the Pearson correlation analysis, the regression was still not significant (P = 0.31).

Tensile strength Width was significantly and positively correlated to D, hole-shape and texturizing, when net square metre weight was used as weighting factor (Table 3), but only D was significant. When texturizing variable was removed, the overall regression declined from 0.91 to 0.85, but significance increased to 0.007. Hole-shape only had a negative impact, but it was not significant (P = 0.16) (Table 3).

### Commercial nets

**Figure 4:**
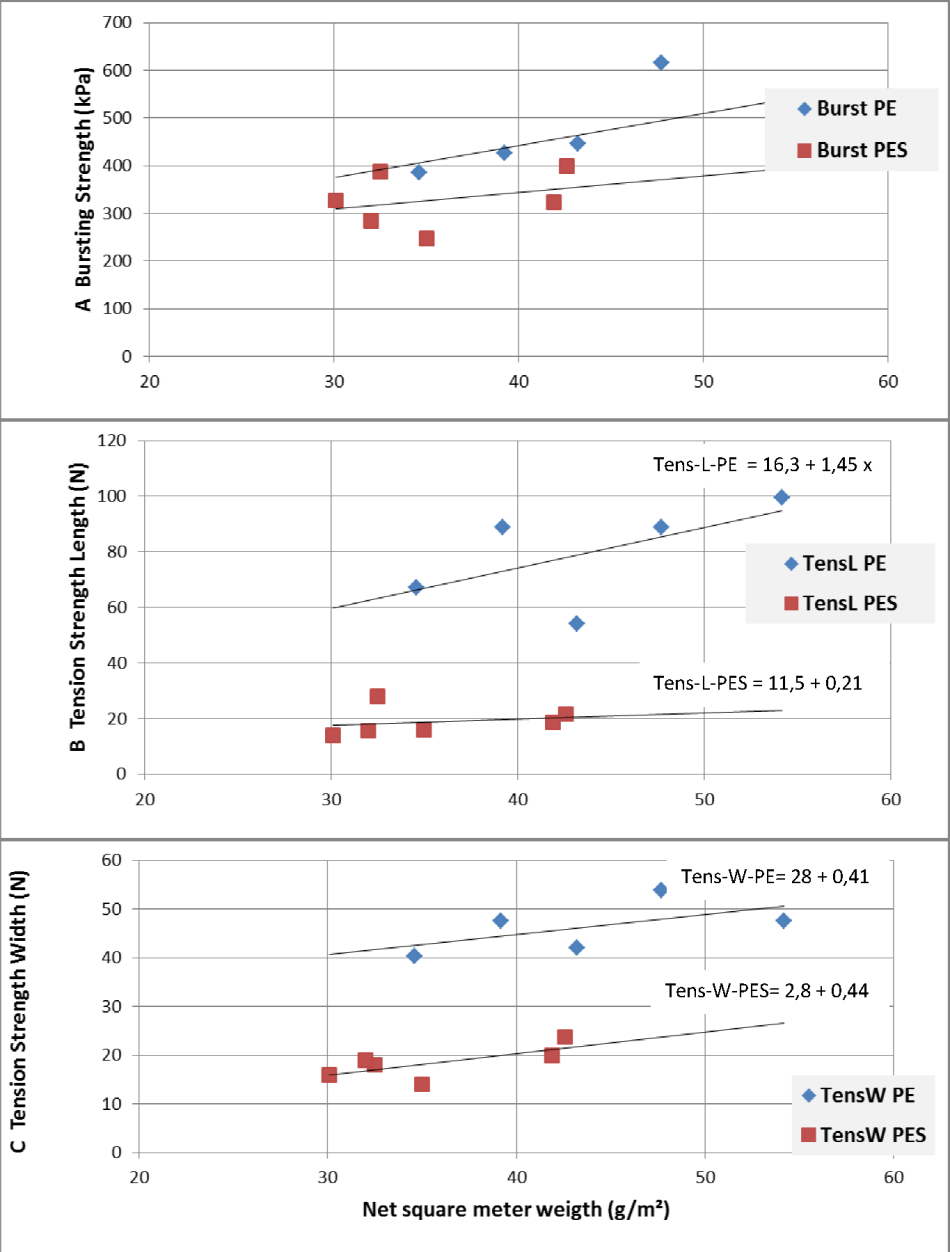
**A. Bursting strength for commercial polyethylene nets and polyester nets**. B. Tension strength direction length for the polyethylene and polyester nets. C. Tension strength width for the polyethylene and polyester nets.

Four commercial PES nets and three commercial PE nets (plus some variations of these) were compared using the same test methods as above (Table 4 and Figure 4). The data were compared in One-Way ANOVA test for significance of differences between means after confirming for homogeneity of variances (Levenes test, [8]). All comparisons made revealed significant differences between means (P < 0,001). Further data comparisons were made with Tukey HSD (Honestly Significant Difference) with significance level 0,05 and the grouping of the means can be seen in Table 4.

**Table 4:** Strength measures of commercial nets

Sample	Weight/m^2 ^(g)	Bursting Strength (kPa at mm)	Tension Strength Grab Hook Length (Newton)	Tension Strength Grab Hook Width (N)	Tension Strength Two Grabs Length (N)	Tension Strength Two Grabs Width (N)
Permanet 2 100 D	42,6	400 at 10 mm ^cd^	28,6^d^	23,7^c^		

Permanet 2 75 D	32,5	388 at 8,7 mm ^d^	28,1^d^	18,0^cd^		

Permanet 2 75 D	30,1	328 at 10 mm ^e^	14,4^d^	15,8^d^	120	110

Permanet 3 Upper part	32,0	285 at 8,8 mm ^ef^	15,5^d^	19,6^cd^		

Permanet 3 Lower Part	41,9	324 at 8,3 mm ^f^	18,7^d^	19,0^cd^		

Interceptor 75 D	35,0	247 at 15 mm ^f^	16,8^d^	14,3^d^	110	83

						

Duranet 255 D	47,7	616 at 12 mm ^a^	88,9^bc^	53,8^a^		

Netprotect 100 D	34,6	385 at 14 mm ^bcd^	66,9^c^	40,3^b^		

Netprotect 118 D	39,2	427 at 15 mm ^cd^	88,9^ab^	47,6^b^	230	150

Olyset 205 D	54,2	468 at 14 mm ^b^	99,4^a^	47,6^b^	290	250

Permanet 3 roof 145 D	43,6	445 at 12 mm ^bc^	53,9^c^	42,0^b^		

The table shows average values of min 5 determinations per net or net part (data from report 8202/2009 and 727/2010 - CITEVE). Values with same letter are not significant different (Tukey test).

Among the PES nets (two Permanet 2, 75 D, one Permanet 2, 100 D, Interceptor 75 D and Permanet 3, upper and lower side apart), bursting strength was lowest for Interceptor, the upper and lower part of Permanet 3, followed by the two Permanet 75 D, and the strongest net was the Permanet 100 D. When all nets are compared (Table 4), there was no difference in tension strength Length between the polyester nets, whereas difference was found in tension strength Width. Interceptor and one Permanet 7 5 D were weaker than the rest, though only significant weaker than Permanet 100 D. Interestingly, the other Permanet 75 D and the Permanet 100 D nearly had the same tension strength Length. If polyester nets are compared as a group alone (data not shown), Permanet 100 D has a significantly higher tensile Strength direction Length than Interceptor, Permanet 3 Upper part and one Permanet 75 D. The other Permanet 75 D and Permanet 3 lower part form a middle group not significantly different from the two other groups. For tension strength Width, Permanet 100D is significantly stronger than all other PES nets, followed by a group including all other PES nets, and the Interceptor that has lower tension strength than all others. Therefore, these analyses show that as for the model nets tension strength generally follows denier. In accordance with this, Permanet 3 sides are significantly weaker in all three measurements than Permanet 100 D, even if the lower part of Permanet 3 has denser knitting and the same weight/m^2 ^as Permanet 100 D. The upper and lower parts of the sides of Permanet 3 sides are made in 75 D yarn, but the knitting of Permanet 3 is a special type that may wake it weaker [7]. The difference in strength measurements between the two 75 D Permanets may be due to different origins of the polyester used for each the nets. The producer of the finished polyester mosquito nets may source the netting material from different polyester manufacturers. As such, the polyester quality is not controlled directly by the LLIN manufacturer, as is the case for the model nets, and may vary.

2 PE nets of the brand Netprotect (one made in 2006 and one in 2010), one Olyset Net, one Duranet and the roof of Permanet 3 (also made out of PE) were compared. Microscopic measurement of the two Netprotect nets showed they had different yarn thickness, 0.123 mm +/- 0,005 (the older) and 0.133+/-0,006 mm (the recent), corresponding to 102 and 121 Denier, respectively. Examination of the Olyset Net showed it had a diameter of 0,173+:-0,010 mm, corresponding to 205 Denier and not the declared 150 Denier. Duranet had a diameter of 0,193 +/-0,014 mm corresponding to approximately 255 Denier and not the declared 150 Denier. The PE roof of Permanet 3 had a diameter of 0,146+/-0,004 mm corresponding to 145 Denier.

Duranet had the highest bursting strength of the polyethylene nets followed by Olyset Net, Permanet 3 roof and Netprotect 115 D and finally Netprotect 100 D. Tear strength Length was highest for the Olyset Net.

The two groups of nets were compared using net weight/m^2 ^as independent variable and Bursting Strength, Tension Strength Length and Tension Strength Width as dependent variables (Figure 4). The slopes and levels were contrasted using the group factor polymer basis: PE or PES. These analyses revealed that bursting strength is significantly dependent on net weight for all nets (P = 0,02), and is higher for the PE nets than for the PES nets, though the two groups overlap (P = 0.09). The slopes for PES nets and PE nets are not significantly different. When Tension Strength Length and Tension Strength Width were analysed as function of net weight, these parameters were significantly dependent on weight (P = 0,02), and the levels of strength were significantly different (P = 0.0004, both cases),with the PE nets being much stronger than the PES nets. The linear regressions are shown in Figure 4.

## Discussion

Nets used in the field become holed already in the first year of use [3,9]. Especially in areas with resistance to the insecticides used in the nets this means a loss of efficacy [1,2]. Many of these holes are tear holes and [9,10] many of these holes are found on the lower part of the net close to the bed frame. Unpublished data from a three-year study in Western Kenya confirmed this. It is, therefore, likely that these holes are due to damage caused by the net getting caught on the bed frame or straw mattress when it is packed away in the morning.

Bursting strength is a measurement that traditionally is used for evaluating net strength. The advantage of this method is that it is not influenced by the different strength of the net in the two directions, Length and Width. On the other hand, the way a net is destroyed in a burst strength test has very little to do with the way nets are damaged as a result of actual use. The bursting test measures the object ability to resist a pressure vertical to the surface. Nets are not destroyed in that way. Contrary to that, the tensile strength determined with one grab and one hook imitates the grip of a hand on the net when pulling it out from the bedside and the net is caught on a pointed object. These data show that tensile strength in the length of the net depends on net weight and yarn type, whereas tensile strength width is correlated to denier, square metre weight and yarn type.

The eight model polyester nets made in the same factory and the variations 100 D or 75 D, texturized or not and knitted in two different way, provide a unique set of data that allows for thorough analysis of impact of these parameters on net strength (Table 1). It is shown that there is a strong interaction between the independent parameters yarn, hole shape and square metre weight (Table 2), so further analysis were carried out as regression analysis using the square metre weight as a weighting factor (Table 3). The analyses of these nets reveal that 100 D nets are stronger than 75 D nets even when compensating for the square metre weight. This means that the yarn weight is the significant variable for strength as the knitting pattern of these nets was the same. 100 D net and 75 D nets had the same number of filaments, 36, which means filaments were thicker in the 100 D net than in the 75 D net. Net strength is thus influenced by the diameter of the microfilaments of the yarn, as suggested by theoretical model of Pan *et al *[11].

It is shown that texturizing weakens the yarn, though not significantly when the weight loss of the yarn due to the texturizing process is taken into consideration. It is also shown that four-sided holes confer greater resistance to tearing than six-sided holes.

The data on commercial nets of the same type (polyester multifilament and polyethylene mono filament, respectively) follow the general rules generated from the systematic eight model net study. Using the tensile strength grab/hook method, the multifilament polyester nets are much weaker than the monofilament polyethylene nets (Table 4 and Figure 4). This is expected since the multifilament yarns burst filament per filament and these filaments are very fine (Netmark report to WHO meeting[12]). When considering bursting strength the differences are smaller and not significant if square metre weight of nets is considered. However, as argued above, this method is not very relevant.

The strongest, commercial PES net was a 100 D net with a square metre weight of 42.6 g/m^2^, 156 mesh - in terms of bursting strength as well as in the two directions of tensile strength in the hook/grab test. The bursting strength of this net was not different from the weakest of the PE net, a 100 D net, mesh 136 and square metre weight 34,6 g/m^2^. However, the PE net had a significantly higher tensile strength.

Two 75 D PES nets were significantly weaker than all other nets, including one other 75 D PES net. This difference is probably linked to a different origin of the polyester, though this net was also slightly heavier (Table 4). Furthermore, Figure 4 shows that the slope of strength parametres to square metre weight is low for polyester. It is generally accepted that 100 D PES nets are not stronger in reality than 75 D nets. It may again be explained by different qualities of polyester used in these nets. When the quality of polyester was controlled in the first 8 study nets, 100 D nets were consistently stronger than 75 D nets.

Permanet 3 was designed to better resistant to wearing [10]. However, the data obtained here does not support this. The lower part and upper part are not significantly different by any measure and significantly weaker than the 100 D net when polyester nets are compared alone. The denser knitting of the lower part did not make it significantly stronger. This confirms the general rule that tensile strength Width is relative to denier.

The commercial PE monofilament nets display a big difference in tensile strength in the two directions. This was much less pronounced for the commercial polyester nets. All commercially available LLIN are constructed in the way that the Width knitting direction of the net textile is the vertical direction of the LLIN. This means that the weakest direction of an LLIN in terms of resistance to tearing is the vertical direction. That is the very way a net is pulled when pulled from a bed frame or mattress.

Polyethylene nets are not made in certain standards as polyester nets. Olyset Net net and Duranet have the thickest yarns and highest square metre weights. The yarn thickness was measured in this study using a micrometre on at least 20 points of the nets. This method is not recommended by the WHO [13]. However, when the product available is a net, it is not possible to get access to hundreds of metres of unbroken yarn to estimate weight as function of yarn length. It is seen that two of the four PE nets had much higher denier than given by the specifications of these nets. In accordance to the rules identified with the tests of the 8 model nets, they therefore have high tensile strength and high bursting strength. The ceiling of Permanet 3 and the Netprotect have lower bursting strength and tensile strength corresponding to their thinner yarns and lower square metre weight. The standard Netprotect is made from a PE polymer mix, according to the WHOPES registration [14], and so is Permanet 3, as indicated in a recent patent application on PE bed nets from the same company [15].

When comparing the three measures for net strength used here for commercial PE and PES nets, PE nets are significantly stronger in tension strength parameters than PES nets, but the nets overlap in burst strength. Figure 4 indicates that this difference increases with increasing net weight; especially for tensile strength Length measured clamp/hook.

The study does not include used nets and it would be highly interesting to see the development of tensile strength after three years of usage. An on-going CDC study of many of the commercial nets will include net strength data. At this time it is not known if they include the tensile strength grab/hook test that we have argued here is the most relevant.

The study of the commercial nets has the weakness that only one or two nets were tested per product. Therefore, the values presented may be extremes. Results obtained from the three PES nets, two of these of the same brand, with the same specification, 75 D, show significant variation how big variation. This clearly warrants further investigation using a greater number of nets.

In conclusion, commercialized, multi-fibre polyester nets are weaker than commercialized mono-fibre polyethylene nets. Analyses of model polyester nets made for this study reveal that the strength of the multifilament polyester net also depends on the diameter of the filaments. Therefore a 75 D net knitted tighter so that it has the same square metre weight as a 100 D net does not have the strength of a 100 D net. However, commercial nets are made of many different qualities of polyester and a 75 D net can be as strong as a 100 D net even if they are from the same producer (Table 4). Yarn thickness in monofilament and filament thickness in polyfilament yarns determine the yarn tensile strength and thus the tensile strength Width of the nets. PES LLIN manufacturers may increase the strength of their products by increasing the diameter of the individual filaments. If the number of filaments is at the same time reduced the nets will maintain their weight. LLIN manufacturers, PE more so than PES, can increase the resistance to tearing of their products by turning the knitting orientation of the net by 90° before sewing. Thus the net will have the Length direction of the textile in the vertical direction providing for a greater resistance to tearing when pulled upwards. Similar strength data should be obtained from used nets.

## Competing interests

The authors are both engaged with the company Intelligent Insect Control. This company has been involved in the development of Permanet versions 2 and 2.5 with Vestergaard-Frandsen and in the development of Netprotect. The company has a direct commercial interest in Netprotect.

## Authors' contributions

OS sent the nets to CITEVE, received the results, made the statistics and wrote the article. RB drafted and edited the article. Both authors have read and approved the final manuscript.
